# Structural Diversity in Galactans From Red Seaweeds and Its Influence on Rheological Properties

**DOI:** 10.3389/fpls.2020.559986

**Published:** 2020-09-10

**Authors:** Marina Ciancia, María Cristina Matulewicz, Rando Tuvikene

**Affiliations:** ^1^ Universidad de Buenos Aires, Facultad de Agronomía, Departamento de Biología Aplicada y Alimentos, Cátedra de Química de Biomoléculas (CIHIDECAR,CONICET-UBA), Buenos Aires, Argentina; ^2^ Universidad de Buenos Aires – Consejo Nacional de Investigaciones Científicas y Técnicas (CONICET), Centro de Investigación de Hidratos de Carbono (CIHIDECAR), Buenos Aires, Argentina; ^3^ Universidad de Buenos Aires, Facultad de Ciencias Exactas y Naturales, Departamento de Química Orgánica, Buenos Aires, Argentina; ^4^ Tallinn University, School of Natural Sciences and Health, Tallinn, Estonia

**Keywords:** sulfated galactans, red seaweeds, carrageenans, agarose, agaran, polysaccharide structure, rheological properties

## Abstract

Galactans are important components of many plant cell walls. Besides, they are the major polysaccharides in extracellular matrixes from different seaweeds, and other marine organisms, which have an acidic character due to the presence of sulfate groups in their structures. In particular, most of the red seaweeds biosynthesize sulfated galactans with very special linear backbones, constituted by alternating (1→3)-β-d-galactopyranose units (A-unit) and (1→4)-α-galactopyranose residues (B-unit). In the industrially significant seaweeds as source of hydrocolloids, B-units belong either to the d-series and they produce carrageenans (as in the order Gigartinales), or to the l-series, and they are sources of agarose and/or structurally related polymers (*i.e.*, Gelidiales, Gracilariales). In both cases, the latter units appear as cyclized 3,6-anhydro-α-galactose in certain amounts, which can be increased by alkaline cyclization of α-galactose 6-sulfate units. Besides, it has been clearly shown that some red algae produce different amounts of both galactan structures, known as d/l-hybrids. It is not yet clear if they comprise both diasteromeric types of units in the same molecule, or if they are mixtures of carrageenans and agarans that are very difficult to separate. It has been reported that the biosynthesis of these galactans, showing that the nucleotide transport for d-galactopyranose units is UDP-d-Gal, while for l-galactose, it is GDP-l-Gal, so, there is a different pathway in the biosynthesis of agarans. However, at least in those seaweeds that produce carrageenans as major galactans, but also agarans, both synthetic pathways should coexist. Another interesting characteristic of these galactans is the important variation in the sulfation patterns, which modulate their physical behavior in aqueous solutions. Although the most common carrageenans are of the κ/ι- and λ-types (with A-units sulfated at the 4- and 2-positions, respectively) and usually in agarans, when sulfated, is at the 6-position, many other sulfate arrangements have been reported, greatly influencing the functional properties of the corresponding galactans. Other substituents can modify their structures, as methyl ethers, pyruvic acid ketals, acetates, and single stubs of xylose or other monosaccharides. It has been shown that structural heterogeneity at some extent is essential for the proper functional performance of red algal galactans.

## Introduction

Many different polysaccharides are found in seaweeds, but they are only a small proportion of the possible theoretical structures. In the course of evolution, these polysaccharides adapted to the environments where the seaweeds are found. Some of them are reserve polysaccharides, degraded to provide energy requirements, and others are constituents of cell walls, but many have functions which at present are not at all well understood (Percival, 1979).

Red algae possess considerably unique morphological and physiological features such as photosynthetic antennae with phycobiliproteins, unstacked thylakoids, floridean starch as cytosolic carbon storage (Woelkerling, 1990; Ho, 2020), cell wall with unique sulfated galactans in addition to cellulose (or other fibrillar polymers) and hemicellulose-like polysaccharides (Popper et al., 2011; Ho, 2020).

**Figure 1:**
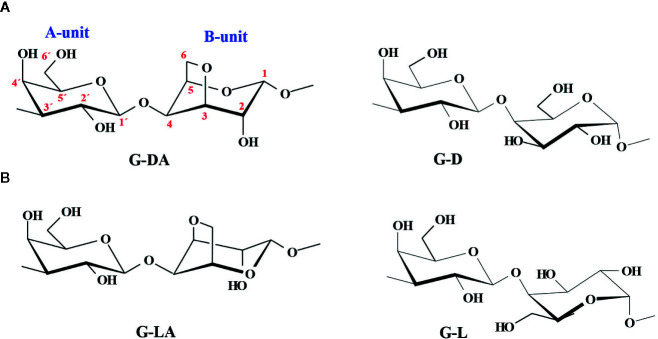
Alternating diasteromeric backbones of sulfated galactans from red seaweeds, named according to the currently accepted nomenclature of Knutsen et al., 1994. **(A)** Carrageenan backbones, **(B)** Agaran backbones. The cyclized agaran backbone (**G-LA**) corresponds to the idealized structure of agarose. The numbering system is shown in red.

Galactans are important components of many plant cell walls. Two types of β-d-galactans are widely distributed in plant cell walls. 4-Linked β-d-galactan chains are important substituents of the backbone of rhamnogalacturonan I (RGI), one of the major polysaccharides in pectins (Caffal and Mohnen, 2009). They are known as Type I galactans, and they may have non reducing terminal arabinose units or short 5-linked arabinofuranose chains linked to C-3 of some of the galactose units, plus minor amounts of other monosaccharides. Type II galactans are a diverse group of short 3- and 6-linked β-D-galactan chains connected to each other by 3,6-linked residues, some of them also contain short arabino-oligosaccharide chains. They are major constituents of arabinogalactan proteins (AGPs), proteoglycans molecules located in Golgi-derived vesicles, the plasma membrane, and the cell wall (Ma et al., 2018). In some cases, they are found in pectins as part of the complex RGI structure (Caffal and Mohnen, 2009). Besides, galactans are the major polysaccharides in extracellular matrixes from different algae, marine invertebrates, and other organisms; these galactans have an acidic character due to the presence of sulfate groups esterifying some of the hydroxyl groups of their monosaccharide components (Pomin, 2009). In particular, most of the red seaweeds biosynthesize sulfated galactans with a backbone that has no equivalent in terrestrial plants, constituted by alternating 3-linked β-d-galactopyranosyl units (A-unit) and 4-linked α-galactopyranosyl residues (B-unit). In the industrially significant seaweeds as source of hydrocolloids, B-units belong either to the d-series and they produce carrageenans (as in Gigartinales), or to the l-series, and they are sources of agarose and/or structurally related polymers (*i.e.*, Gelidiales, Gracilariales). In both cases, the latter units appear as cyclized 3,6-anhydro-α-galactose in a certain amount (**Figure 1**). Although the chemistry of sulfated galactans from these red seaweeds is fairly well studied, their biosynthesis is still under investigation (Usov, 2011).

The currently accepted model of biosynthesis is thought to occur in three sequential steps involving three main classes of enzymes (Rees, 1961a; Rees, 1961b; Hemmingson et al., 1996; Genicot-Joncour et al., 2009). During the first step, galactosyltransferases catalyze chain elongation *via* the transfer of activated galactose residues. Secondly, galactose residues are sulfated by sulfotransferase enzymes. Finally, galactose 6-sulfurylases or sulfohydrolases produce ciclyzation of galactose 6-sulfate moieties to 3,6-anhydro derivatives.

Initial biosynthesis steps would occur from: i) Fructose 6-phosphate derived from Calvin cycle, or ii). An existing carbohydrate pool of floridean starch and floridoside. Floridean starch is the main reserve polysaccharide of red macroalgae, which is degraded during respiration to form UDP-d-glucose *via* UDP-d-glucose pyrophosphorylase (Lluisma and Ragan, 1999; Goulard et al., 2001). The enzyme UDP-galactose 4-epimerase, subsequently interconverts UDP-d-glucose to UDP-d-galactose (Frey, 1996). Various studies in terrestrial plants and algae support its involvement in cell wall polysaccharides synthesis as supplier of UDP-d-galactose (Joersbo et al., 1999; Seifert et al., 2002; Barber et al., 2005; Rösti et al., 2007). In red algae UDP-d-galactose was shown to be the most abundant nucleotide sugar (Manley and Burns, 1991; Goulard et al., 1999).

A coordinated activity of at least two galactosyl transferases is suggested to form the alternating 3-linked β- and 4-linked α-linkages, making up the polymer backbone of carrageenans. Even though these reactions have been shown to take place in the Golgi apparatus, none of these enzymes have been isolated (Genicot-Joncour et al., 2009; Ficko-Blean et al., 2015).

For agarans, UDP-d-galactose and GDP-l-galactose are required, being the galactose enantiomers derived from d-glucose and d-mannose, respectively, which are supplied by different biosynthetic pathways.

Production of GDP-l-galactose is catalyzed by the enzyme GDP-mannose 3’,5’-epimerase (GME, EC 5.1.3.18). GME catalyzes two steps of epimerization reactions at 3- and 5-positions of GDP-d-mannose giving the nucleotide sugar GDP-l-galactose (Wheeler et al., 1998; Major et al., 2005). The biosynthesis of UDP-d-galactose would happen as described previously.

Sulfated galactan heterogeneity depends largely on sulfotransferases. Sulfation occurs at different positions of the galactan backbone that are specific for a certain algal group, and each position requires specific sulfotransferases. This step is suggested to occur in the Golgi apparatus (Genicot-Joncour et al., 2009; Ficko-Blean et al., 2015).

In the final step in galactan biosynthesis, the sulfated carrageenan is believed to be transported to the cell wall where the cyclization of the α-galactose 6-sulfate units to 3,6-anhydrogalactose, catalyzed by a galactose-6-sulfurylase, takes place. This remains the only step in algal carrageenan biosynthesis that has been biochemically demonstrated to date (Wong and Craigie, 1978; Genicot-Joncour et al., 2009; Ficko-Blean et al., 2015). A similar cyclization step was reported for agarans (Lee et al., 2017). This ring closure, which involves change of the *^4^C_1_* conformation of the former units to *^1^C_4_* of the latter units, leads to helix formation of galactans in aqueous environments (Tuvikene et al., 2008).

Agar was reported to accumulate in the cell walls, embedded in a structure of fibers of crystallized cellulose, constituting the amorphous matrix of the cell wall. An intermediate form of low molecular weight sulfated agar is secreted by the Golgi apparatus and it is deposited in the cell wall, where it enzymatically polymerizes and desulfates, giving mostly into agarose rendering the agar industrially produced as gelling agent (Arminsén and Gálatas, 2009).

There are many red seaweeds which biosynthesize both carrageenan and agaran types of galactans. It is not clear yet whether carrageenan and agaran disaccharidic repeating units are in the same molecule, or they constitute separate molecules which form aggregates that until now are very difficult to break. Many studies about them appeared for decades, but it was during the nineties that it became evident that many of the most common and industrially relevant seaweed species of carrageenophytes and agarophytes produce small to considerable quantities of galactans of the other group, containing their diastereometric repeating units. This is an interesting finding, from the biological point of view, because it would indicate that carrageenophytes, as well as agarophytes have the enzyme machinery to biosynthesize both types of galactans. This issue has been reviewed before, and it will not be considered with further detail (Stortz and Cerezo, 2000; Takano et al., 2003; Ciancia and Cerezo, 2010; Usov, 2011).

The majority of structural studies on polysaccharides have been carried out on extracts or mixtures obtained from certain red seaweed. In many cases, the chemical structures are only the average of all the molecules present which is often a family of polydisperse heteromolecules, all built up on the same general plan, but differing in their fine details. However, in some studies, extensive fractionation protocols involving anion exchange chromatography or precipitation of the polysaccharides with quaternary ammonium salts were carried out. The detailed study of these fractions brought light into these fine structural characteristics. Modifications in the structure of these macromolecules would enable the seaweed to fulfill different functions.

The aims of this review are to correlate the chemical structures of the galactan backbones and their substitution patterns with the phylogenetic relationship of the different red algal groups and their functional properties.

## Carrageenans

Carrageenans from the most important carrageenophytes have been thoroughly studied. These galactans have structures based on a linear chain of alternating 3-linked β-d-galactopyranosyl residues (A-units) and 4-linked α-d-galactopyranosyl residues (B-units), the latter unit is often found mostly cyclized, giving the 3,6-anhydro-derivative (**Figure 1A**).

Variation of carrageenan structures are mainly due to their sulfation pattern and/or the presence of 3,6-anhydrogalactose units. Carrageenans from the most important carrageenophytes are rarely pyruvylated or methoxylated, and they have low degree of branching, if any. Nevertheless, other carrageenophytes show complex substitution patterns.

**Figure 2:**
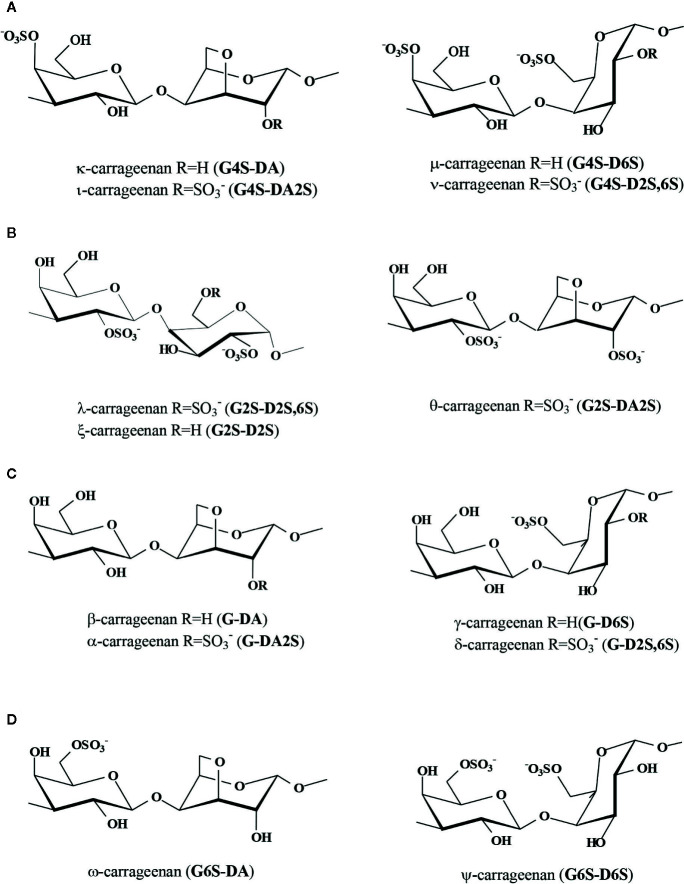
Structures of carrageenans of the different families: **(A)** κ-family, **(B)** λ-family, **(C)** β-family, and **(D)** ω-family. In parenthesis, diads named according to the nomenclature of Knutsen et al., 1994.

Classification of carrageenans takes into account the sulfation pattern of the A-unit (Stortz and Cerezo, 2000). The κ-family comprises 4´-sulfated polysaccharides, while the λ-family includes those with sulfate at the 2´-position. Less important are the β-family, in which the A-unit is not sulfated, and the ω-family with sulfation at the 6´-position of this unit (**Figure 2**).

In relation to the B-units, in carrageenans of the κ-family, the cyclized units predominate, giving rise to κ- or ι-carrageenans, with disaccharidic repeating units **G4S-DA** and **G4S-DA2S**, respectively, according to the accepted shorthand terminology system developed by Knutsen et al. (1994) (**Figure 2A**). Some seaweeds of the family Solieriaceae biosynthesize major quantities of carrageenans with clear predominance of one of these structures. For example, *Kappaphycus alvarezii* produces mainly κ-carrageenans (Anderson et al., 1973; Estevez et al., 2004), while *Eucheuma denticulatum, E. perplexum*, and *E. kraftianum* produce major amounts of ι-carrageenans (Anderson et al., 1973; Bellion et al., 1983; Santos, 1989), as well as *Solieria filiformis* and *Agardhiella subulata* (Murano et al., 1997). Another seaweed, *Hypnea musciformis* (Cystocloniaceae), is also known to produce large amounts of κ-carrageenan (Greer et al., 1984; Cosenza et al., 2014). It has been shown that differences in carrageenans between generic phases (gametophytes and tetrasporophytes) in most families of the order Gigartinales, as Solieriaceae, Cystocloniaceae, Furcellariaceae are negligible (Doty and Santos, 1978; Usov, 2011).

However, seaweeds from the Gigartinaceae and Phyllophoraceae give different carrageenan structures in both macroscopic life stages (Ciancia and Cerezo, 2010). The haploid individuals, that usually predominate, produce major amounts of polysaccharides in which κ- and ι-disaccharidic units form blocks of the same molecule (van de Velde, 2008), with predominance of the former in seaweeds of Gigartinaceae, like *Chondrus crispus* and *Gigartina skottsbergii* (Matulewicz et al., 1989; Ciancia et al., 1993a; Falshaw et al., 2001; Guibet et al., 2008), while in the Phyllophoraceae, like those of the genus *Gymnogongrus* (Estevez et al., 2008; Pérez Recalde et al., 2016), *Stenogramma interruptum* (Cáceres et al., 2000), and *Ahnfeltiopsis flabelliformis* (Kravchenko et al., 2016), major quantities of the latter structure are found. However, recently, a κ/ι-carrageenan obtained from *Mastocarpus pacificus* (Phyllophoraceae) showed predominance of κ-structure (Kravchenko et al., 2020). These carrageenan κ/ι-hybrids are commercially known as κ-2 (Falshaw et al., 2001).

In addition, water extracts of all these seaweeds have variable amounts of the biological precursors of κ- and ι-carrageenans, which are usually known as µ- and v-carrageenans, respectively (**Figure 2A**), and they can be transformed in the former by treatment with alkaline solutions (Bellion et al., 1983; Matulewicz et al., 1989; Ciancia et al., 1993b; Stortz and Cerezo, 1993; Cáceres et al., 2000; Jouanneau et al., 2010). Hence, when these seaweeds are extracted with alkali or submitted to alkaline pretreatments, precursor units are not found in considerable amounts (Azevedo et al., 2013).

**Figure 3:**
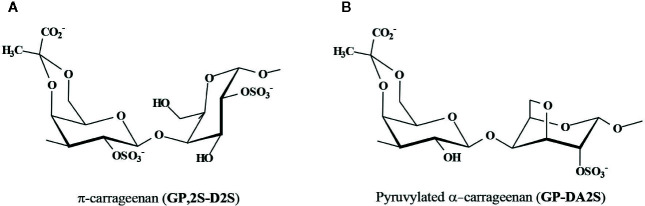
Major pyruvylated carrageenan structures. **(A)** π-carrageenan, a pyruvylated carrageenan of the λ-family, found in some tetrasporophytes from the Gigartinaceae, and **(B)** Pyruvylated α-carrageenan found in major quantities in some Placentophoraceae Areschouglaceae, Acrotylaceae, and Solieriaceae, and also in minor amounts in some Phyllophoraceae.

Besides, the diploid macroscopic phase (tetrasporophytes) of seaweeds from the Gigartinaceae and Phyllophoraceae biosynthesizes carrageenans of the λ-family (**Figure 2**), which mostly occur as non-cyclized structures, mainly λ-carrageenan, **G2S-D2S,6S** (McCandless et al., 1973; McCandless et al., 1982; Matulewicz et al., 1989; Stortz and Cerezo, 1993; Falshaw and Furneaux, 1994; Stortz et al., 1994; Falshaw and Furneaux, 1995; Falshaw and Furneaux, 1998; Falshaw and Furneaux, 2009). From *Iridaea undulosa* (Stortz et al., 1994) and *Rhodoglossum gigartinoides* (as *Gigartina lanceata*) (Falshaw and Furneaux, 1998) almost “pure” λ-carrageenans were obtained. However, those from tetrasporophytes of other species of the Gigartinaceae, as *G. grandifida, G.divaricata*, and *Chondracanthus teedei* were more complex, as they comprised important amounts of ξ-carrageenan (**G2S-D2S**) and π-carrageenan (**GP,2S-D2S**) (**Figures 2B** and **3A**) (Falshaw and Furneaux, 2009; Pereira and van de Velde, 2011). Pyruvate was detected in the λ-carrageenans from these species (Falshaw and Furneaux, 1995; Falshaw and Furneaux, 1998; Falshaw and Furneaux, 2009), as **GP,2S** in considerable amounts (6%–13%). In addition, some of these carrageenans showed important amounts of **G2S,6S** (16%–22%) and **D2S** (21%–26%) (Falshaw and Furneaux, 1998; Falshaw and Furneaux, 2009). At first, detection of **G2S,6S** units indicates that these carrageenans should not be classified in one of the defined carrageenan families. Nevertheless, taking into account the absence of cyclized B-units, as well as the high degree of sulfation at the 2-position of the B-units (**D2S** + **D2S,6S**), and that they are produced by tetrasporophytes of species from some Gigartinaceae, we consider that they could be included provisionally in the λ-carrageenan group. In fact, these carrageenans also include major quantities of the typical λ-carrageenan structural units. It is important to note that these species with complex structures have also morphological differences with other Gigartinaceae, which biosynthesize λ-carrageenans as the only significant galactan structure (Falshaw and Furneaux, 2009).

Sulfohydrolases which act at the polymer level producing 3,6-anhydro-d-galactose with the stoichiometric release of sulfate have been identified in both diploid and haploid macroscopic individuals from *Chondrus crispus* (Gigartinaceae). However,λ-carrageenan is not susceptible to their action and, it is thought to inhibit their activity (Wong and Craigie, 1978; Ficko-Blean et al., 2015). The fact that the alkaline cyclization is notably slower for λ-carrageenan, than for ν-carrageenan, suggested that, at least in these conditions, the sulfation position on the A-unit is determinant for the cyclization reaction of the B-unit (Ciancia et al., 1993b).

However, cyclized λ-carrageenans, known as θ-carrageenans (**G2S-DA2S**) (**Figure 2B**), are major constituents of seaweeds of the genus *Callophyllis* (Kallymeniaceae) (Falshaw et al., 2005; Rodriguez et al., 2005). The d-galactose-6-sulfurylase responsible for the conversion of λ-carrageenan to θ-carrageenan has not yet been characterized (Ficko-Blean et al., 2015).

β-Carrageenan (**G-DA**) (**Figure 2C**) is produced in important quantities by *Furcellaria lumbricalis* (Furcellariaceae), *Tichocarpus crinitus* (Tichocarpaceae), and *Betaphycus gelatinus* (as *Eucheuma gelatinum*) (Solieriaceae), mostly as β/κ-hybrid carrageenan (Greer and Yaphe, 1984; Santos, 1989; Knutsen et al., 1990; Barabanova et al., 2010; Correc et al., 2012), and it was possible to isolate it in a pure form from *Betaphycus gelatinus, B. speciosus* (as *Eucheuma gelatinum, E. speciosa*, Solieriaceae), and *Endocladia muricata* (Endocladiaceae) with low yield (Renn et al., 1993).

Highly pyruvylated carrageenans of the β-family were isolated from six species of the genus *Callophycus* (Placentophoraceae); these galactans consisted predominantly of the repeating disaccharide 4’,6’-*O*-(1-carboxyethylidene)carrabiose 2-sulfate (**GP-DA2S**) and minor amounts of the α-carrageenan repeating unit (**G-DA2S**) (**Figure 2C**) (Chiovitti et al., 1997; Falshaw et al., 2003). Although found in several different red seaweeds, this pyruvylated structure, which belongs to the β-family, was not named with a Greek letter yet (**Figure 3B**). Pyruvate was also detected in minor amounts in the carrageenan from *Stenogramma interruptum* and *Coccotylus truncatus* (Phyllophoraceae) (Miller, 1998; Cáceres et al., 2000; Tuvikene et al., 2009).

Chiovitti et al. (1998a) reported for the carrageenan from *Sarconema filiforme* (Solieriaceae) disaccharide repeating units of the ι- and α-types, some of the latter being substituted with pyruvate. In three *Erythroclonium* species (Areschouglaceae), the carrageenans showed a substitution pattern of the repeating disaccharide units **G4S-DA2S**, **G-DA2S**, the 6´-*O*-methylated counterparts, and **GP-DA2S**. Significant amounts of unsubstituted, 4-linked galactopyranose and small amounts of 4-linked 3-*O*-methylgalactopyranose and terminal glycosyl residues were also detected (Chiovitti et al., 1998b). Highly 6´-*O*-methylated ι-carrageenans (**G4S,6M-DA2S**) containing 3-*O*-methylgalactose and pyruvate were isolated from two species of the genus *Rhabdonia* (Areschouglaceae) (Chiovitti et al., 1996). It is important to note, that in the case of carrageenans from *R. coccinea*, the degree of 6´-*O*-methylation was particularly high (31 mol % of **G6M**). Later, the same group determined the structure of carrageenans from *Claviclonium ovatum* (Acrotylaceae, Gigartinales), which were nearly idealized 6´-*O*-methylated ι-carrageenans (Chiovitti et al., 2004). All these red seaweed families that are phylogenetically close and far from the most important carrageenophytes (Verbruggen et al., 2010; Guiry and Guiry, 2020) biosynthesize carrageenans with very complex substitution patterns, which include structures of the β- and κ-families.

Carrageenan devoid of 3,6-anhydrogalactose and sulfate (**G-D**) has not been found in Nature (Usov, 2011).

Carrageenans of the ω-family (**G6S-DA**) (**Figure 2D**) are not widely distributed. They were isolated in important quantities from *Phyllophora nervosa* (Phyllophoraceae). This result is noteworthy, as seaweeds of this genus usually produce κ/ι-carrageenans as major sulfated galactans (Usov and Shashkov, 1985). Mollion et al. (1986, 1987, 1988) isolated a homogeneous carrageenan with this structure from *Rissoella verruculosa* (Rissoelaceae). This carrageenan was obtained by alkaline treatment and further fractionation with a 0.3 M KCl solution where it remained soluble. The authors tried several strategies in order to find the theoretical precursor of ω-carrageenan (Mollion et al., 1987), called ψ-carrageenan (**G6S-D6S**) (**Figure 2D**), but they did not succeed. Hence, they proposed that in this case, the biosynthetic pathway in which sulfated carrageenans derive from the nonsulfated, β-carrageenan could occur. One approach to the biosynthesis of these polymers is the observation that a carrageenan structure is often bound to its precursor within the same molecule, forming chains of what is usually called a partially cyclized carrageenan. So, the evidence to propose this was the fact that they were not able to separate the ω- and β-carrageenan structures, but they found β/κ-carrageenan hybrids. As far as we know, ω-carrageenans were not detected in significant quantities in other carrageenophytes.

The carrageenan from *Phacelocarpus peperocarpos* (Phaceolocarpaceae) comprised major quantities of **G4S,6S-DA** (Liao et al., 1996). This galactan cannot be included in any of the carrageenan families described so far. To the best of our knowledge, this is the only species of this family studied until now regarding its carrageenan structures. Further work could derive in the definition of a new carrageenan family.

**Figure 4:**
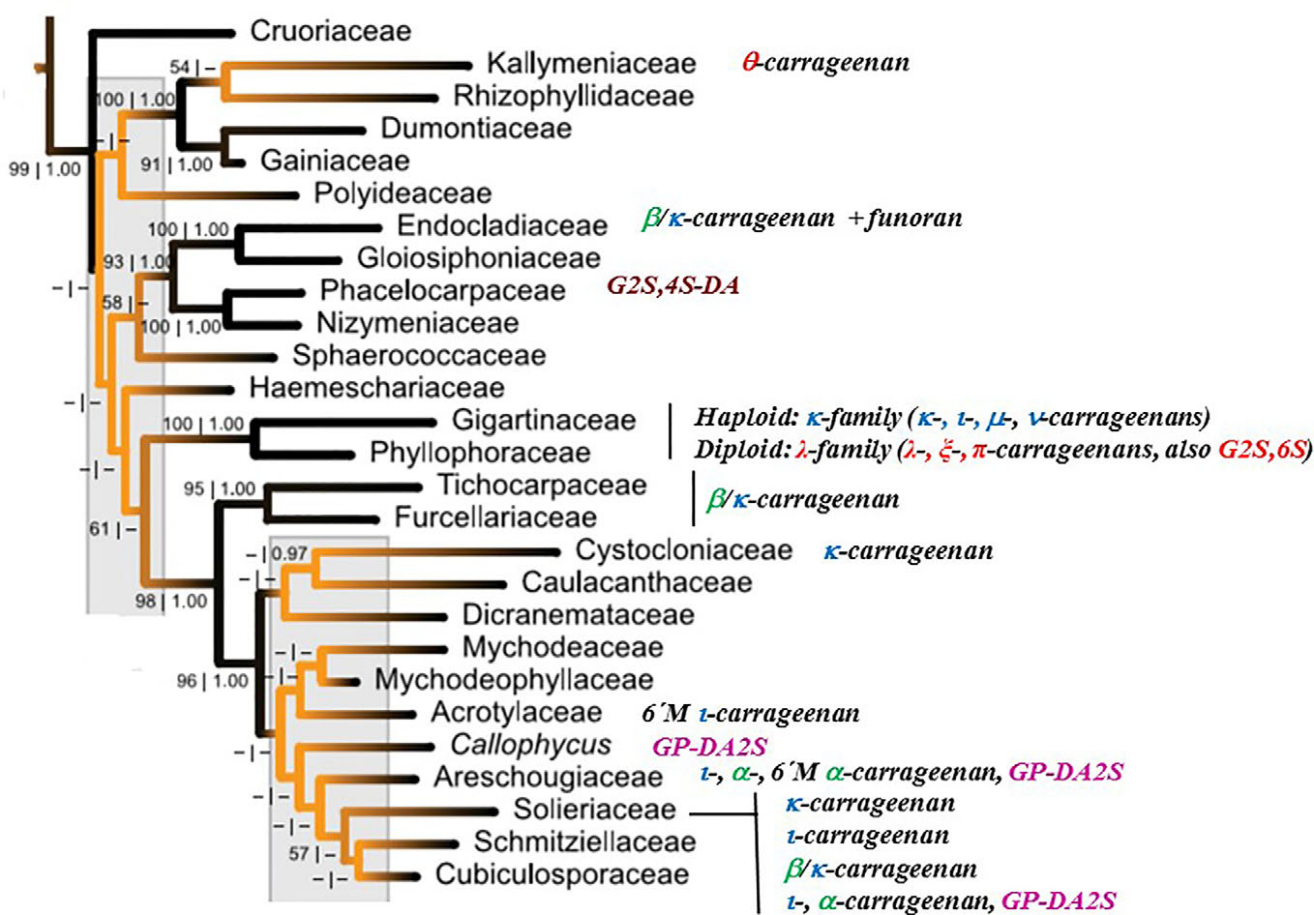
Major carrageenan structures produced by the different taxonomic families of the Gigartinales, based on the red algal tree of life reported by Verbruggen et al., 2010. Families comprising major amounts of galactans of the D/L-hybrid group were not included.

**Figure 4** shows the major carrageenan structures produced by the different taxonomic families of the Gigartinales, based on the red algal tree of life reported by Verbruggen et al. (2010).

## Agarans

Agarans are sulfated galactans constituted by alternating 3-linked β-d-galactose units and 4-linked α-l-galactose units **(** **Figure 1B** **)**. The 4-linked units can be partially or wholly converted to 3,6-anhydro forms by enzymatic elimination of sulfate from the 6-position. According to the initial substitution pattern, this conversion may afford the neutral polysaccharide agarose (**Figure 1B**) or agarose derivatives. Agarose is the major component of the products industrially obtained mainly from seaweeds of the orders Gelidiales, Gracilariales, and Ahnfeltiales (Arminsén and Gálatas, 2009), and known as “agar” or “agar-agar”.

Due to the complexity and diversity of their substitution pattern (Murano, 1995; Usov, 2011), it has not been possible to classify agarans into ideal “repeating units”. In fact this backbone is usually masked by different substituents:

**-** The 3-linked and 4-linked units can carry sulfate groups, methoxyl groups, and/or β-d-xylopyranosyl side chains at different positions.
**-** The 3-linked units can carry 4-*O*-methyl-α-l-galactopyranosyl groups at the 6´-position.
**-** The 3-linked units can carry pyruvate cyclic ketals at the 4´- and 6´-positions.

Actually, only the term agarose has a strict chemical sense, whereas other polysaccharides of the group are usually termed according to the algal species from which they were isolated (Damonte et al., 2004). The basic structure of the agaran backbone sulfated at the 6-position of the B-unit, **G-L6S**, according to the nomenclature of Knutsen et al. (1994), is known as porphyran because it is biosynthesized by seaweeds of the genus *Porphyra* (Bangiaceae) in important amounts (Morrice et al., 1983). This structure can be converted in agarose by enzymatic or chemical cyclization of **L6S** to give **LA**, in a similar way as that indicated for carrageenans.

A classification taking into account the sulfation pattern of the A-units is possible, leading to the following agaran groups.

### a) G Group Comprises Agarans in Which the A-Unit Is Not Sulfated

It is based on a polysaccharide with **G-L** and **G-LA** backbones. Taking into account the diversity of the substitution patterns in agarans, the following backbones are also included in this group: **G-L6S**, **G6M-L6S**, **G-L2M6S**, **G6M-LA**, **G6M-LA2M**, **G-LA2M**, **GP-LA**, single stubs of 4-*O*-methyl-l-galactose linked to the 6´-position of G [**G6(L4M)**] of agarose or porphyran repeating units.

The **G-LA** repeating unit of agarose, is present in the orders Gelidiales (*Pterocladiella capillaceae*, Pterocladiaceae; *Gelidium rex*, *Gelidium corneum* (as *G. sesquipedale*), Gelidiaceae), Anhfeltiales (*Anhfeltia plicata* and *A. tobuchiensis*, Anhfeltiaceae), and Gracilariales *(Gracilariopsis longissima* (as *Gracilaria verrucosa*), *G. cervicornis, G. blodgettii, Crassiphycus crassissimus* (as *Gracialaria crassissima*), *G. gracilis, Crassiphycus birdiae* (as *Gracilaria birdiae*), *Gracilariopsis hommersandii*, Gracilariaceae) in major quantities (Matsuhiro and Urzua, 1990; Errea and Matulewicz, 1994; Murano, 1995; Errea and Matulewicz, 1996; Freile-Pelegrín and Murano, 2005; Truus et al., 2006; Rodríguez et al., 2009; Souza et al., 2012; Rodríguez Sánchez et al., 2019; Zhang et al., 2019; Martínez-Sanz et al., 2020).

The **G-L6S** repeating unit was found in the agarans (porphyrans) from *Porphyra capensis* and *Porphyra* *haitanensis* (now *Pyropia* *haitanensis*) (Bangiales, Bangiaceae) (Zhang et al., 2004; Zhang et al., 2005). From the room temperature water extract from *Pyropia columbina* (as *Porphyra columbina*), a fraction rich in **G6M-L6S** (54%) was isolated; the remaining material corresponded to partially 6´-*O*-methylated agarose [**G(6M)-LA**]. The alkali treated derivative gave agarose and its 6´-*O*-methylated derivative in molar ratio 3:7 (Noseda et al., 2000). The **G6M-LA** and **G6M-LA2M** repeating units were detected in *Gracilaria arcuata* (Tako et al., 1999). Falshaw et al. (1998) studied the agars from nine species of the genus *Curdiea* (Gracilariaceae), and detected highly methylated agaroses, some of these species biosynthesized **G-LA2M** as major repeating unit, while in others **G6M-LA** or **G6M-LA2M** predominated. The **G-L2M6S** repeating unit was reported in an agaran from fresh water *Bostrychia moritziana* (Youngs et al., 1998). The repeating unit containing pyruvate **GP-LA** was found in the agarans of *Hydropuntia edulis* (as *Gracialria edulis*) (Falshaw et al, 1999), as well as in some other *Gracilaria* species (Murano, 1995). **G6(L4M)** was detected in the agarans of *Gracilariopsis longissima* (as *Gracilaria verrucosa*) (Karamanos et al., 1989) and *Gracilaria tikvahiae* (Craigie and Jurgens, 1989).

Water soluble agarans obtained after removal of agarose from *Gracilariopsis lemaneiformis* (as *Gracilaria lemaneiformis*) and *Gelidium amansii* showed considerable amounts of **G-L6S** (16.2 and 15%, respectively). In addition, for *G. lemaneiformis*, no methylated derivatives were found and only small amounts of **G6S** and **G4S** were detected, while for *G. amansii*, these galactose derivatives were present in minor quantities, but also minor amounts of **GP** and **LA2M** were detected (Wang et al., 2016).

**Figure 5:**
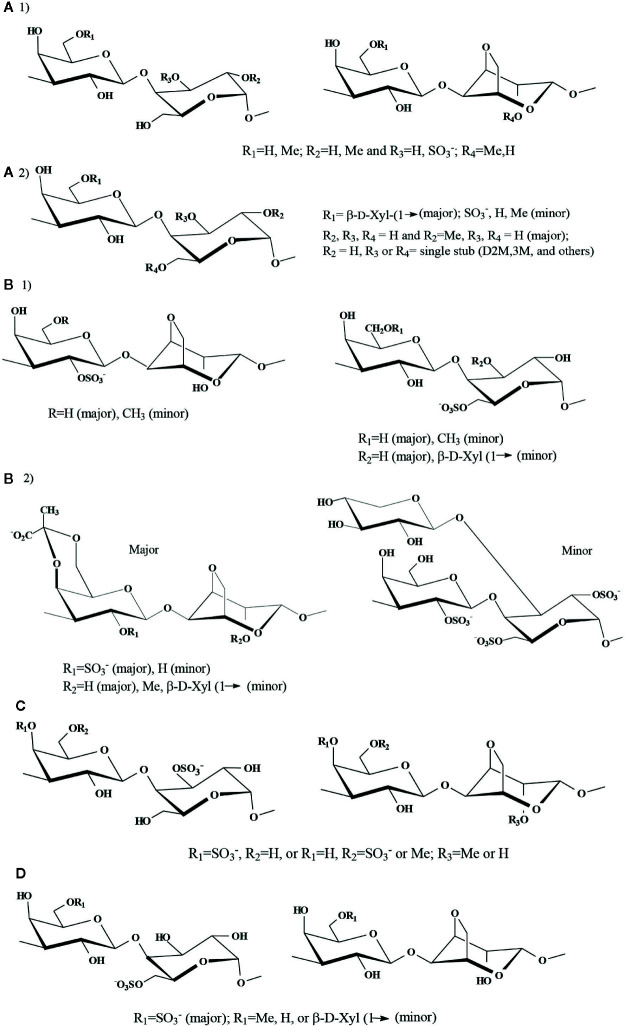
Proposed major disaccharidic reapeating units of highly substituted agarans. **(A)** **G group**: 1) Agaran from *Georgiella confluens* (Kolender and Matulewicz, 2002), 2) Agaran (corallinan) from *Lithothamnion heterocladum* (Navarro et al., 2011); **(B) G2S group**: Agarans from: 1) *Laurencia obtusa* and 2) *L. filiformis* (Canelón et al., 2014); **(C)** **G4S group:** Agaran from *Bostrichya montagnei* (Duarte et al., 2002); **(D)** **G6S group**: Agarans from *Polysiphonia nigrescens* (Prado et al., 2008).

The **G-L** diad, the basic repeating unit of an agaran, was found in native *Georgiella confluence* (Ceramiales, Callithamniaceae), in which **G-L3S** was also detected in important amounts. The structure got further complicated by partial methylation at the 6´-position of **G** and at the 2-position of **LA** units; other structures were present in minor amounts (Kolender and Matulewicz, 2002) [**Figure 5A** **(1)**].

The agaran-like backbones (**G-L**) from red seaweeds of the Corallinales (Corallinaceae), known as corallinans should be included in this group. They showed essentially a very similar substitution pattern between each other: the **G** residue bore single stubs of β-d-xylose or methoxyl groups at the 6´-position, and methoxyl groups and sulfate occupied the 2- or 3-position of the B-unit (Cases et al., 1992; Cases et al., 1994; Takano et al., 1996; Usov et al., 1997; Navarro and Stortz, 2008; Navarro et al., 2011). However, besides these common features, **G6(L4M)** units were found in *Corallina officinalis* (Cases et al., 1994); the presence of **LA**, **LA2M**, and **LA2S** replacing the **L** residues, together with single stubs of 2,3-di-*O*-methyl- and 3-*O*-methyl-d-galactose, and 3-*O*-methyl-l-galactose were detected in *Jania rubens* (Navarro and Stortz, 2008). *Lithothamnion heterocladum* (Corallinales, Lithothamniaceae) biosynthesizes an agaran-like polysaccharide with the general characteristics described before for the Corallinaceae. However, substantial amounts of single stubs of 2,3-di-*O*-methyl-d-galactose and 4-linked α-l-galactose units glycosylated at the 6- or 3-positions also appeared (Navarro et al., 2011) [**Figure 5A** **(2)**].

### b) G2S Group Comprises Agarans in Which the A-Unit Is Sulfated at the 2´-Position

It is based on polysaccharides with **G2S-L6S** and **G2S-LA** backbones. This agaran group is found in major amounts on algae of the Rhodomelaceae (Ceramiales). These diads were found in agarans from *Laurencia* species, as *L. nipponica* (Usov and Elashvili, 1991)*, L. obtusa* and *Laurencia gracilis* (as *L. filiformis*) (Canelón et al., 2014) [**Figures 5B** **(1)** and **(2)**], *L.* *dendroidea* (Ferreira et al., 2019), and others; and also in the related species *Acanthophora spicifera* (Duarte et al., 2004)*, Chondria macrocarpa* (Furneaux and Stevenson, 1990), and *Palisada flagellifera* (Ferreira et al., 2012). In some of these species, the repeating unit **GP2S-LA** was also detected in considerable quantities (*L. gracilis* (as *L. filiformis*)*, A. spicifera, C. macrocarpa*, and *P. flagellifera*). Each of the agarans of these species showed particular substitution patterns in the B-units: In *L. nipponica, P. flagelifera, C. macrocarpa, L. obtusa*, and *L. dendroidea*, the presence of β-d-xylose at the 3-position precluding cyclization of the **L6S**, was found. In the agarans of *L. nipponica, P. flagelifera*, and *L. gracilis* (as *L. filiformis*), **LA2M** was found, but in the latter case, **LA2S** and **LA2X** were also detected.

### c) G4S Group Comprises Agarans in Which the A-Unit Is Sulfated at the 4´-Position

Sulfation at the 4´-position is seldom observed in galactans of the agaran type, contrary to what is found in carrageenans. Major amounts of agarans of this group were obtained from *Bostrychia montagnei* (Rhodomelaceae, Ceramiales), comprising major amounts of **G4S**, **G6S**, and **G6M**. The B-units were mainly **LA2M, LA** and **L2S** (Duarte et al., 2002) (**Figure 5C**). Another seaweed of the same family, *Odonthalia corymbifera*, contained a substituted agarose with major quantities of **G6M,4S-LA** (Usov, 2011).

Repeating units corresponding to this group were also detected in *Hydropuntia eucheumatoides* (as *Gracilaria eucheumatoides*) (**G4S-LA2M**) and *Gracilaria pseudoverrucosa* (**G4S-LA**) (Lahaye et al., 1989).

In *Griffithsia antarctica* (Wrangeliaceae, Ceramiales) (Miller, 2003a) and in *Gracilariopsis hommersandii* (Gracilariaceae, Gracilariales) (Rodríguez Sánchez et al., 2019) major amounts of **G4S-LA** and **G4S-L6S**, and lesser quantities of **G4S-L3S** were found. The agaran from *Haraldiophyllum crispatum* (as *Myriogramme denticulata*) and *Hymenena palmata* f*. marginata* (Delesseriaceae, Ceramiales) consisted mainly of one diad, **G4S-L2S,3S** (Miller, 2001; Miller, 2005).

### d) G6S Group Comprises the Agarans in Which the A-Unit Is Sulfated at the 6´-Position

The presence of repeating units **G6S-LA** and **G6S-L6S** was reported in the agarans from *Vertebrata fucoides* (as *Polysiphonia nigrescens*) (Prado et al., 2008) (**Figure 5D**), *P. morrowii* (Usov et al., 1983; Usov and Ivanova, 1987), *Melanothamnus strictissimus* (as *Polysiphonia strictissima*), *P. abscissoides* (Miller and Furneaux, 1997), *P. atterima* (Miller, 2003a) (Rhodomelaceae, Ceramiales). Sulfation at the 6´-position was also detected in important levels in *Pterosiphonia pennata* (now known as *Xiphosiphonia pennata*) (Miller, 2003a), *Lophurella hookerina* and *L. caespitosa* (Miller, 2002), *Streblocladia glomerulata* (Miller and Furneaux, 1997), and *Cladhymenia oblongifolia* (Miller and Blunt, 2000), the five species belonging to Rhodomelaceae, Ceramiales. In a galactan fraction obtained recently from *Polysiphonia senticulosa* by anion exchange chromatography, eluting with 2.0 M NaCl, in addition to the characteristic 6´-sulfation pattern**, GP** and **LA2X** units were detected in important quantities (Zhao et al., 2017).

In addition, **G6S-LA** was the only structure in a fraction from the cold water extract from *Gloiopeltis furcata* (Endocladiaceae, Gigartinales) obtained in 20% yield regarding the dry seaweed (Hu et al., 2012), and it was also found in major amounts in this and other *Gloipeltis* species (*G. complanata* and *G. tenax*), with minor amounts of **G6M-LA, G6S-LA2M, G6S-L6S**, and **G-LA**. These agarans are usually called funorans (Tuvikene et al., 2015a).

The polysaccharides of the Corallinales described in (a) also bear a certain degree of sulfation at the 6´-position. Thus, these agarans belong to the **G** and **G6** groups, being an example of **G/G6S** hybrids, analogously to what is observed in β/κ-carrageenans from *Rissoella* species (Mollion et al., 1986; Mollion et al., 1987).

Agarans from *Hymenena palmata, H. variolosa*, and *Acrosorium* *decumbens* (Ceramiales, Delesseraceae) were reported to be highly sulfated. They contained **G2S,4S,6S-L2S,3S** and even totally sulfated repeating disaccharides (Miller, 2005). Highly sulfated agaroses containing pyruvate were isolated from two species of *Sarcodia, S. montagneana* and *Sarcodia grandifolia* (as *S. flabellata*) (the order Plocamiales) (Miller, 2003b). However, these assignments were made based on methylation analysis and IR and ^13^C NMR spectroscopy, and should be confirmed. As the author mentions undermethylation could be misinterpreted as high substitution; determination of the sulfate content was not carried out.

In these sections, the major galactans produced by each of the mentioned seaweeds were considered. Most of these red algae comprise variable quantities of other polysaccharides, biosynthesized in small amounts that are believed to modulate the properties of their amorphous cell wall matrixes.

## Structure and Functionality of Red Seaweed Galactans

The gelling or viscosifying nature of seaweed galactans is essential for the vital functions of red algae. Fine organization of the polysaccharide chains to form microscopic gel networks makes these polymeric assemblies effectve as molecular sieves and gives seaweed the required flexibility to withstand marine conditions. High water-binding capacity of the gel matrices helps seaweed to maintain its hydrated state during periodic dry periods, which is especially important for the algae inhabiting intertidal zones.

Due to the large structural variations, wide range of rheological properties can be obtained on the basis of the galactans deriving from red seaweeds. This makes these polysaccharides useful in various applications to provide the required texture for food and non-food systems. Although the galactans belonging to agar and carrageenan families are closely related, the functional properties of these polysaccharides are notably different.

### Carrageenan Type Polysaccharides

All carrageenans are soluble in water at room temperature when converted into their Li^+^- or Na^+^-forms, whereas the presence of K^+^-, Ca^2+^-, or Mg^2+^-ions impairs the solubility, especially for κ-type carrageenans, making them soluble only at elevated temperatures. The solubility of λ-carrageenan is the least affected by the cationic composition and the galactan remains soluble in cold water.

In favorable cationic environments, κ-carrageenan shows the highest gelling ability among carrageenans, yielding slightly opaque, rigid gels with poor freeze-thaw stabilities, whereas ι-carrageenan affords weak, elastic gels which are more stable towards freezing and thawing cycles. λ-carrageenan forms solutions with very high viscosities. The non-gelling behavior has also been noted for α-carrageenan extracted from *Catenella nipae* which has reported to show the ability to form viscous solutions (Zablackis and Santos, 1986). Similar rheological properties have been observed for θ-carrageenan prepared by alkaline modification of λ-carrageenan originating from the tetrasporophytic form of *Gigartina skottsbergii* (Doyle et al., 2010).

Sulfation at the 2-position of β-d-galactopyranose residues generally impairs the gel-forming ability of algal galactans by preventing the chains to form helixes (Morris et al., 1978). Studies on some oversulfated carrageenan samples prepared by chemical modification processes have revealed an ability to form a weak gel for a specific carrageenan type bearing three sulfate ester groups per disaccharide repeating unit (**G4S,6S-DA2S**), whereas the tetra-sulfated derivative (**G2S,4S,6S-DA2S**) containing an additional sulfate ester group at the 2-position of β-d-galactopyranose moiety did not form a gel under any circumstances (Thành et al., 2001). Poor gelling ability has been found to be inherent for ω-carrageenan from *Rissoella verruculosa* (Mollion et al., 1986). Weak gelling ability have been reported for β-carrageenan samples (~150 g/cm^2^ at 1%) obtained from *Betaphycus gelatinus, B. speciosus* (as *Eucheuma gelatinum, E. speciosum*) and *Endocladia muricata* (Renn et al., 1993; Thành et al., 2002).

The 3,6-anhydrogalactose is essential for the formation of a gel network and its content usually correlates well with the gelling ability of the galactan. The presence of precursor units (e.g. μ-, ν-, and λ-carrageenans) not bearing cyclized B-units, commonly leads to decrease in gel strength values. The gel melting and coil-to-helix transition temperatures are influenced by the presence of precursor units in lesser extents (van de Velde et al., 2003).

### Agar Type Polysaccharides

Agarose completely dissolves in water at 97–100°C. 1.5% solutions of commercial agaroses commonly gel between 32–43°C and the formed opalescent gels typically melt above 85°C. The thermal hysteresis between gelation and melting is very high, almost always exceeding 45°C and can even reach above 70°C. Melting temperature of agarose gels as well as the gel strength values are highly dependent on the molecular weight, while gelling temperatures are less affected (Watase and Nishinari, 1983).

The setting and melting temperature of agar gels are notably influenced by the methoxylation degree of the polysaccharide. The natural methoxylation at the 6´-position of β-d-galactopyranose residues is particularly responsible for elevated gelling temperature, while the methoxy groups at the 2´-position, often arising from chemical methylation process, tend to lower the gel setting temperature (Miller et al., 1994; Arminsén and Gálatas, 2009). Although the melting temperature of agarose gels can be raised by methoxylation, significant increase takes place only by double methoxylation pattern, i.e. by co-occurrence of methoxy groups at the 6´-position of β-d-galactopyranose and at the 2-position of 3,6-anhydro-α-l-galactopyranose residues (Falshaw et al., 1998). Notable examples are agars extracted from *Curdiea obesa* and *C. coriacea*, both having more than 90% of their 6´-position of **G** and 2-position of **LA** units methoxylated. These samples were reported to melt at 120–121°C and 112–113°C, respectively, at 1% concentration (Falshaw et al., 1998). Melting points around 100°C have been observed for 1% agars from *Gracilaria arcuata* v. Snackeyi with 80%–90% methoxylation at both 6´-position of **G** and 2-position of **LA** residues (Falshaw et al., 1999).

Agarose is among the most potent gelling substances known. The storage modulus (G’) of quality agaroses at 1.5% can remain around 50,000 Pa, which is notably higher than for κ-carrageenan gel systems containing K^+^- and Ca^2+^-ions (~20,000 Pa). Nevertheless, it has been recently shown that at some specific counter-ion compositions κ-carrageenan can form stronger gels than is commonly observed for agaroses—the G’ of 1.5% κ-carrageenan gel system containing equal amounts of Rb^+^- and Sr^2+^-ions and no free salts was found to be 81,000 Pa, whereas the combination of K^+^- and Sr^2+^-ions yielded the gel with G’ = 48,000 Pa at 15°C (Robal et al., 2017).

Gel strength of electrophoresis grade agaroses commonly remains in the range of 1,000–3,000 g/cm^2^ for 1.5% and between 600–1,600 g/cm^2^ for 1.0% polysaccharide concentration. Association of agarose helixes is favored by the 3,6-anhydrogalactose residues (Sousa et al., 2013) and any modification in a α-l-galactopyranose moiety preventing the occurrence or formation of this 3,6-anhydro derivative has a detrimental effect on the gelling ability. Rheological properties of agars can be thus often notably enhanced by cyclization reaction in alkaline medium (Noseda et al., 2000; Freile-Pelegrín and Murano, 2005) or by the action of sulfohydrolases (Shukla et al., 2011). Gel strengths of native agars commonly remain many times lower than the pure agarose fractions obtained after alkaline treatment process. Agaroses with very high gelling ability (2200 g/cm^2^ at 1.0%) have been obtained from *Gracilaria dura*, whereas native agars from this species form significantly weaker gels (< 300 g/cm^2^) (Meena et al., 2007).

Methoxylation at the 6´-position of **G** and the 2-position of **LA** units does not notably impair the gelling ability of agarose and gel strength values as high as 1560 g/cm^2^ (at 1%) have been observed for highly methoxylated preparations (Falshaw et al., 1998). However, the agarose with abundant **G6M** have shown to yield less turbid gels with slightly higher G’ values than the low methoxy content agarose with very similar molecular weight characteristic (Brenner et al., 2019). Sulfation of agarose molecules, irrespectively of the substitution pattern, always leads to impaired gelling ability. It has been shown that the sulfate group at the 2´-position of β-d-galactopyranose residue destabilizes double-helical conformation as hydroxyl group in this location forms internal hydrogen bond within the double helix (Rees, 1969; Montaño et al., 1999). Also pyruvate and branched building blocks containing xylose or 4-*O*-methyl-α-l-galactopyranose residues diminish gel-forming ability. The frequent structural modifications with full 6-sulfation of α- or β-galactopyranose residues, do not show gelling properties under low co-solute conditions. Porphyran occurring in *Porphyra* and *Pyropia* species has been reported to exhibit pseudoplastic properties with flow behavior index decreasing with increasing concentration (Koo et al., 2007). For chemically modified agars (e.g. carboxymethylated, acetylated samples), gel setting, gelling temperatures and gel strength values decrease with increasing substitution degree of the galactan (Cao et al., 2014; Xia et al., 2014).

Essentially pure funoran (**G6S-LA**) fraction from *Gloiopeltis* species is fully soluble in cold water (Tuvikene et al., 2015a), but does not form a gel under low ionic strength conditions. At high cation concentration, aggregation of the polysaccharide chains and subsequent gelation takes place due to shielding of the sulfate groups. For funorans, Ba^2+^-ions have the highest gel promoting ability, yielding 1.5% gels with G’ >25,000 Pa in 0.12 mM BaCl_2_ solution (Tuvikene et al., 2015b). Such Ba^2+^ containing gels show turbid and opalescent characteristics, very similar to those of agarose, whereas the other alkaline earth and alkali metal ions lead to funoran gels with high transparency. The gel formation of such systems is slow enough to allow quick introduction of the salts promoting gelation, thus making it possible to completely avoid the heating steps during the preparation of such galactan gels.

### Hybrid Galactans

The conformational irregularities introduced by μ- or ν-carrageenan repeating units impede the helix formation and affect the gelling process of κ- and ι-carrageenans (Vilén et al., 2010). It has been shown that ι-carrageenan preparations completely lose their gelling ability at ν-carrageenan amounts exceeding 20 mol% (van de Velde et al., 2002). However, small levels of structural irregularities are required for proper gel forming ability of the galactans. Findings on ι-/ν-carrageenan hybrids have confirmed that the gelling ability of the preparations containing 3% of ν-repeating units was notably higher than for the essentially pure ι-carrageenan samples (van de Velde et al., 2002).

Pure κ- and ι-carrageenans show different rheological properties compared to κ-/ι-carrageenan hybrids. Higher level of κ-repeating units leads to enhanced gelling ability (van de Velde, 2008). Nevertheless, the functional properties of κ-/ι-carrageenan hybrids have been reported to remain fairly consistent when the molar ratio of κ-to ι-repeating units remains in the range of 1.2–4.0 (Bixler et al., 2001).

Rheological properties of hybrid carrageenans are also influenced by the mode of distribution of different structure types along the polysaccharide chains (van de Velde et al., 2001; Villanueva et al., 2004; Souza et al., 2011). Furcellaran, which is a κ-/β-carrageenan hybrid with about 50 mol% of the κ-repeating units desulfated, behaves similarly to κ-carrageenan, although smaller amount of counter-ions is required to achieve optimal gel strength for this polysaccharide (Bird et al., 1991). Its conformational transition temperatures are, however, a bit higher and the gel strength values slightly lower than commonly observed for κ-carrageenan (Zhang et al., 1991; Robal et al., 2017). Functional similarity between furcellaran and κ-carrageenan arises from the random distribution of κ- and β-repeating units along the galactan chains. This ensures a regular distribution of charges and permits good gelling properties. The κ-/β-carrageenan hybrids with more block-wise distribution of the repeating units show lower gelling ability (Correc et al., 2012).

Studies on the artificial mixtures of agarose and κ-carrageenan have revealed that the gelation of such blends takes place through the formation of a molecular interpenetrating gel network with no evidence of simple phase separation (Amici et al., 2002).

### Factors Affecting the Gel Properties

#### The Effect of Counter-Ions and Co-Solutes

All carrageenans, except pure β-carrageenan, need a certain amount of cationic counter-ions (commonly Na^+^-, K^+^-, Ca^2+^-, Mg^2+^-ions) to balance the negative charge of the sulfate ester groups of the polysaccharide. If the level of galactan-bound cations is not enough, degradation of the polysaccharide takes place through removal of sulfate groups, acidification and autohydrolysis processes (Robal et al., 2017).

Specific counter-ions in certain amounts are required to maintain the functional properties of carrageenans. Cationic species which favor the gelling or viscosifying properties of specific galactan types (especially K^+^- and Ca^2+^-ions) generally show higher affinities towards the galactan matrices and are often found in larger quantities also in seaweed biomass (Tuvikene et al., 2008; Tuvikene et al., 2009). Gelation of κ-carrageenan is promoted by K^+^-, Rb^+^- and Cs^+^-ions, whereas Mg^2+^-, Ca^2+^-, and Sr^2+^-ions enhance the formation of ι-carrageenan gels. λ-carrageenan forms a gel-like system in the presence of Fe^3+^-ions (Running et al., 2012). The κ-carrageenan systems in which the negative charges of sulfate ester groups are balanced only by the gel promoting monovalent cations, are characterized by poor gelling abilities (Chen et al., 2002; Robal et al., 2017). For the maximum gelling ability, the co-presence of mono- (K^+^, Rb^+^ or Cs^+^) and divalent (Mg^2+^, Ca^2+^, Sr^2+^ or Ba^2+^) ions is needed. By combining the ratio of K^+^- and Ca^2+^-ions in κ-carrageenan preparations, 1% gels with G’ varying between 70-43000 Pa have been obtained (Hermansson et al., 1991). The effect of Ca^2+^-ions is based on the formation of intra or intermolecular cross-bridges between the sulfate ester groups in carrageenan chains (Nickerson et al., 2004).

The addition of CaCl_2_ increases the gelling and gel melting temperatures and also the thermal hysteresis of κ-carrageenan (Liu and Li, 2016). The stiffness of the gels commonly increases with increasing KCl, whereas the gels prepared in KCl solutions are commonly more transparent than these gelled in the presence of CaCl_2_ (Nguyen et al., 2014). Li^+^- and Na^+^-ions enhance solubility and impair gelling ability of carrageenans. The rheological properties of κ-carrageenan gels can be thus controlled by varying the ratio of K^+^- and Na^+^-ions in the system (Mangione et al., 2005). It has been shown that 1% κ-carrageenan gels containing both 20 mM KCl and 200 mM NaCl exhibit heterogeneous structure and smaller mesh size than is typical for κ-carrageenan systems (Lorén et al., 2009). The aggregation of κ- and ι-carrageenan helixes is prevented by some anionic substances, especially by I^–^ and SCN^–^ions, which notably impair the gelling ability of such polysaccharides (Zhang et al., 1991; Takemasa and Nishinari, 2004).

The gelling ability of agarose is somewhat affected by the presence of anions. NaCl has a weak stabilizing effect, whereas NaBr, NaNO_3_, NaSCN and Na_2_SO_4_ destabilize the agarose gel and facilitate syneresis (Piculell and Nilsson, 1989; Singh et al., 2009). Gelling ability of agars bearing sulfate groups is enhanced in the presence of some cationic species. Agars with 36% of **LA** and 4.5% of sulfate (as **G4S**) extracted from *Gracilaria edulis* showed enhanced gelling ability, from 190 g/cm^2^ to 350–400 g/cm^2^ (at 1.5%) by the addition of Na^+^-, K^+^- or Ca^2+^-ions (Villanueva and Montaño, 1999). Addition of high levels of low-molecular sugars (glucose, sucrose) into agarose systems leads to reduced crosslinking in the polysaccharide network (Deszczynski et al., 2003). It has been also noted that the influence of molecular weight on gel properties is reduced with the addition of sucrose (Normand et al., 2003). Freezing of agar gels leads to significant syneresis after thawing and the process has been thus employed in a traditional agar manufacturing process. Freeze-thaw stability of the gels can be improved with the addition of sucrose or by rapid freezing through pressure-shift technique, which has proven to be more effective in preserving the quality of the frozen agar gels compared to the conventional freezing at -20°C or even at -80°C (Fuchigami et al., 2006).

#### The Effect of Other Factors

The presence of 3,6-anhydrogalactopyranose residues in agars and carrageenans leads to increased flexibility of the galactan chains and allows larger contraction of the random coil structure (Therkelsen, 1993). Helical conformation of κ-carrageenan is stiffer than for ι-carrageenan, which is suggested to be the underlying feature yielding brittle κ-carrageenan and elastic ι-carrageenan gel networks (Schefer et al., 2015). During the gel forming process of algal galactans, the initial aggregation involves longer polysaccharide chains whereas shorter chains form aggregates on further cooling (Dai and Matsukawa, 2012; Zhao et al., 2013). In the case of κ-carrageenan, the structure of the aggregates has been shown not to depend on the molecular weight, but the level of aggregation was found to decrease with lowering of the length of the polymer chains (Meunier et al., 2001).

Properties of the galactan gels are notably influenced by the cooling rate during the gelling process, higher gelling abilities are obtained at slow cooling whereas the enthalpies are not much affected by the speed of cooling (Moritaka et al., 2007). The storage modulus of 1% κ-carrageenan has been found to be 3 times higher for the gel obtained by cooling at 0.5°C/min compared to this formed at 1.5°C/min (Hermansson et al., 1991). The thermal history greatly influences the junction zone formation. It has been shown that at slow cooling, large junction zones form in κ-carrageenan systems, whereas many small junction zones appear at fast gelling rates (Iijima et al., 2007). For the agar gel systems, it has been evidenced that at slow cooling the extent of aggregation of double helices is larger than for agar gels obtained by rapid quenching of the solution. It has been shown that the agar gels formed by rapid cooling to 5°C melted at 12–13°C lower temperatures compared to those prepared by cooling at 1°C/min (Mohammed et al., 1998). Furthermore, the formation of network structures shifts towards higher temperatures with increasing agarose concentrations (Mohammed et al., 1998).

## Concluding Remarks

Sulfated galactans are produced in important yields by many different red marine macroalgae. These galactans are divided in two groups, carrageenans and agarans, which have diasteromeric structures (**Figure 1**). Although both groups have many important common structural features, they have different determinant structure-rheological properties relationships. Industrial hydrocolloids from the agaran group are neutral polysaccharides (agarose), while industrially significant galactans of the carrageenan group regarding their rheological properties (κ-, κ/ι- β/κ-carrageenans, and λ-carrageenan in a lesser extent) are sulfated. This fact determined that sulfated polysaccharides of the carrageenan group were thoroughly studied, while in the case of agarophytes, most investigations tried to get rid of them. Hence, the knowledge about structural details of sulfated galactans from agarophytes is not as wide as that of carrageenans. In addition, structures of sulfated agarans are more complex in some cases.

There is a very well established classification of carrageenans in families named with Greek letters according to the sulfation pattern on the A-units (**Figure 2**). Although a similar classification for agarans would not be so useful due to the relative importance of the different groups (the majority of the agarans belong to the **G group**), defining different agaran groups according to the sulfation at the A-unit clearly shows that most of the agarophytes rich in agarose or its precursor, porphyran, belong to certain orders (Gelidiales, Gracilariales, Ahnfieltiales, and Bangiales), while most of the groups containing sulfate esters in the A-unit belong to the Ceramiales. In this review, seaweeds comprising d/l-hybrid galactans were not treated in depth, because it is not yet clear if they comprise both diasteromeric types of units in the same molecule, or if they are mixtures of carrageenans and agarans that are very difficult to separate by the currently available methodologies, which include ion exchange and gel permeation chromatography and those comprising precipitation of the polysaccharides with quaternary ammonium salts as the most employed techniques. In the case of carrageenophytes, differential precipitation of carrageenans of the κ-family with potassium chloride of increasing concentrations, rendered water soluble fractions enriched in d/l-hybrid galactans (Ciancia and Cerezo, 2010; Usov, 2011). A lot of work is still needed in this field. Both possibilities could happen, and it would be of great taxonomic and also practical importance to know the real structures of these galactans in relation to the algal groups from which they are obtained.

Most of the agarophytes of the **G group** biosynthesize major amounts of agarose, which is not sulfated, but it can have variable amounts of methoxyl groups and other substituents. These agarophytes also produce variable amounts of the precursor units of agarose, **G-L6S**, which is found in different quantities depending of the seaweed and of the extraction conditions. There is also a group of agarans (corallinans) that cannot be cyclized because they lack sulfate at the 6-position, but can have variable quantities of sulfate at the 6´-position, which are extracted from the Corallinales.

While carrageenans are produced in major quantities only by algae from the Gigartinales, agarans are biosynthesized by red seaweeds from different orders, namely, Gelidiales, Gracilariales, Ahnfeltiales, Bangiales, Ceramiales, Corallinales, and others only studied in a few cases. An important exception to this asseveration was found for galactans from the family Endocladiaceae (Gigartinales), which were shown unequivocally to comprise in the case of *Endocladia muricata* β/κ-carrageenans by ^13^C NMR spectroscopy and optical rotation (Renn et al., 1993), but major amounts of agarans, called funorans, in the case of *Gloipeltis* species (Hu et al., 2012; Tuvikene et al., 2015a). However, d/l-hybrids were reported previously for some *Gloiopeltis* species (Takano et al., 1998), although predominance of agarans was proved. Also for *Endocladia muricata*, besides β/κ-carrageenans, 6% agarobiose was detected (Whyte et al., 1985).

Seaweeds from the order Ceramiales produce major amounts of agarans with complex structures that comprise an important degree of sulfation on the A-unit. The sulfation pattern of these algae is very variable and greatly depends of the taxonomic family. The general substitution pattern of these agarans is further complicated by the presence of variable amounts of other substituents. However, their complexity is also dependent of the algal group. For example, agarans from *Polysiphonia* species are not so complex, compared with those of *Laurencia* species, and other related algae, both genera belong to the Rhodomelaceae, so in this case it is possible to find chemtoxonomic relationships below the taxonomic family level. Besides, some species of the Gracilariales have also shown particular sulfation patterns on the A-unit, although most of them belong to the **G group**. All these agarans are usually not industrially relevant by their rheological properties, but some have remarkable biological activities.

In some of these galactans, the acidic nature is given not only by the presence of sulfate groups, but also by the presence of 3-linked 4’,6’-*O*-(1-carboxyethylidene)-β-D-galactose residues as A-units. Interestingly, there are two different very well defined groups of carrageenans having pyruvic acid ketals (**Figure 3**). One of them is the group of galactans from tetrasporophytes of some Gigartinaceae, which comprise important amounts of π-carrageenans, a non-cyclized carrageenan, sulfated at the 2´and 2-positions. The other group obtained from seaweeds of the Placentophoraceae Areschouglaceae, Acrotylaceae, and Solieriaceae, has pyruvic acid ketals in a cyclized carrageenan structure, only sulfated at the 2-position. Both polymers should be very different regarding their conformation in water solutions. In agarans, pyruvic acid ketals were also found in certain algal groups, as some species of *Gracilaria* (Gracilaraceae, Gracilariales) and *Laurencia* (Rhodomelaceae, Ceramiales).

While biosynthesis pathways for carrageenans and agarans have been proposed, experimental knowledge on algal biosynthetic enzymes remains still very limited. Moreover, the biological significance of the structural variability of these cell wall sulfated galactans is not understood at all. The challenge is not only to understand their biosynthesis, but also related metabolic pathways in relation to their evolution, and in adaptation to different environments. These studies could help to modulate their functional properties in culture.

## Author Contributions

MC and MM analyzed and discussed the sulfated galactan structures. RT analyzed and dicussed the functional properties of sulfated galactans. MC and MM programmed the review structure. MC, MM, and RT integrated the whole analysis and wrote the manuscript.

## Funding

Financial support was provided by the Estonian Research Council grant PUT1406 and by the Tallinn University ASTRA project “TU TEE—Tallinn University as a promoter of intelligent lifestyle” financed by the European Union European Regional Development Fund 2014-2020.4.01.16-0033. MC and MM receive financial support from the National Research Council of Argentina (CONICET) (PIP 11220130100762CO 2014-2016, PIP 112-2015 01-00510, PU-E 2016 22920160100068CO) and the University of Buenos Aires (UBACYT 2018-2021, 20020170100347BA, 20020170100292BA).

## Conflict of Interest

The authors declare that the research was conducted in the absence of any commercial or financial relationships that could be construed as a potential conflict of interest.
